# The clinical experience of early skin-to-skin contact combined with non-nutritive comfort sucking in mothers of preterm infants: a qualitative study

**DOI:** 10.1186/s12884-023-05581-x

**Published:** 2023-04-24

**Authors:** Liling Li, Futing Ji, Yuejue Wang, Li Wang, Ling Yu, Xi Wu, Tianchan Lyu, Yalan Dou, Yun Cao, Xiao-jing Hu

**Affiliations:** 1 Children’s Hospital of Fudan University, the National Children’s Medical Center, 399 Wan Yuan Road, Shanghai, 201102 People’s Republic of China; 2 Research Unit of Early Intervention of Genetically Related Childhood Cardiovascular Diseases(2018RU002), Chinese Academy of Medical Sciences , Beijing, China

**Keywords:** Neonatal intensive care unit, Very low birth weight infant, Skin-to-skin contact, Non-nutritive comfort sucking

## Abstract

**Background:**

In most areas of China, mothers typically do not participate in early care of preterm infants in NICU. This study aims to examine the early experience of mothers of preterm infants participating in skin-to-skin contact combined with non-nutritive comfort sucking in China.

**Methods:**

This qualitative research study used one-on-one, face-to-face, semi-structured in-depth interviews. Eighteen mothers who participated in early skin-to-skin contact combined with non-nutritive comfort sucking were interviewed in the NICU of a tertiary children’s hospital in Shanghai between July and December 2020. Their experiences were analyzed using the inductive topic analysis method.

**Results:**

Five themes about skin-to-skin contact combined with non-nutritive comfort sucking were identified, including alleviation of maternal anxiety and fear during mother infant separation, reshaping the maternal role, promotion of active breast pumping, enhances the mother’s willingness to actively breast feed and building the maternal confidence in baby care.

**Conclusion:**

Skin-to-skin contact combined with non-nutritive comfort sucking in the NICU can not only enhance the identity and responsibility of the mother’s role, but also provide non-nutritive sucking experience for promoting the establishment of oral feeding in preterm infants.

## Background

Preterm infants are prone to sucking, swallowing and respiratory coordination disorders due to immature brain development and immature reflexes, which eventually can result in oral feeding difficulties [1, 2]. Non-nutritive comfort sucking on an empty breast is helpful to the development of feeding skills. It not only provides sucking and swallowing training for infants and shortens the transition time from tube feeding to full oral feeding in premature infants [3], but also promotes lactation in mothers and enhances the emotional bond between mothers and their premature infants. Kangaroo mother care (KMC) refers to early, immediate, continuous or intermittent skin-to-skin contact with parents of preterm or low birth weight infants in the early stages of hospitalization or after discharge, while ensuring the normal temperature of preterm infants [4, 5]. This promotes neural development in preterm infants as well as lactation in the mother and breastfeeding [6, 7]. Skin-to-skin contact is the main component of KMC [8]. Studies have shown that skin-to-skin contact between the mother and infant promotes lactation [9]. Literature describing parents’ experience of KMC reported that parents feel closer to their infants and are more comfortable with their parenting, but are also afraid of hurting their infants during KMC [10–13]. It is currently reported that compared with the parents of healthy full-term infants, parents of infants in the NICU experience emotional and psychological challenges, with significantly increased incidence of depression, anxiety, and post-traumatic stress disorders [14–17].

However, in most areas of China, the neonatal intensive care unit (NICU) is not open to parents. For some NICUs, parents can only see their infants shortly before discharge. For some other NICUs, parents can visit their infants once a week for a short time. Mother-infant separation increases anxiety in mothers and infants [18, 19]. Encouraging parents to participate in infant care is important for optimizing the infant’s health outcomes. The mother’s experience in skin-to-skin contact combined with non-nutritive comfort sucking remains unknown. It is critical to further examine the experience of parents participating in infant care as well as the benefits of parent-infant relationship for the development of the NICU care plan.

Therefore, the purpose of this study was to examine the experience of early skin-to-skin contact and non-nutritive comfort sucking of mothers of hospitalized preterm infants by qualitative research in China. Our findings will provide a reference for NICUs to include mothers in early skin-to-skin contact combined with non-nutritive comfort sucking to promote improvement of clinical outcomes in preterm infants and the feelings of parents.

## Study methods

### Design and setting

This qualitative study was designed as an in-depth interview. Semi-structured interview guide were used to conduct one-to-one face-to-face interviews with mothers of preterm infants in the NICU. The purposive sampling method was used in this study.

The study was carried out at a tertiary children’s hospital in Shanghai. The NICU was designed as nine large rooms with many infants. There are two family rooms in the unit. More than 2,000 high risk infants including more than 400 very low birth weight infants admitted to the unit every year. Two lactation consultants were available in the unit.

### Participants

Recruitment took place between July and December 2020. The mothers of preterm infants hospitalized in the NICU were eligible if meeting the following criteria: (1) maternal age over 20 years without delivery experience; (2) able to fluently communicate in Mandarin Chinese; (3) the gestational age of the mothers was < 30 weeks and birth weight of the infants was < 1500 g; (4) infant hospitalized in the NICU for more than one week and still admitted at the time of interview; (5) maternal involvement in skin-to-skin contact combined with non-nutritive comfort sucking more than 4 times. After the withdrawal of mechanical ventilation, the infant would be assessed for 12 to 24 h. When the infant’s condition was stable, the mother would be invited to enter the NICU to receive a combination of skin-to-skin contact and non-nutritive comfort sucking. Clinical nursing specialists were responsible for including these eligible parents based on the inclusion and exclusion criteria. Before skin-to-skin contact, the mother pumped and expressed breastmilk for about 15 min. Nurses assisted the mother about skin-to-skin contact process and carried out non-nutritive comfort sucking (sucked on empty breast) training for 5 to 10 min. Skin-to-skin contact would be carried out for 1 h at least once a day until discharge, and monitoring should be done at the same time.

### Data collection

According to the purpose of this study and previous reports, the interview guide consisted of four open-ended questions developed by the authors and finalized after discussion by five experts. Four of five experts were neonatal clinical nurse specialists and one was a neonatologist. The clinical nursing specialists are trained in breastfeeding, kangaroo care, and family center care education, and are currently engaged in clinical nursing and education training.

The interview guide was used to conduct a preliminary interview with two mothers of preterm infants participating in skin-to-skin contact combined with non-nutritive comfort sucking. Then, it was modified and determined according to the interview process and results. Finally, the modified interview guide was used as a guide to explore mothers’ experiences during participation in skin-to-skin contact combined with non-nutritive comfort sucking (Table 1). Cues and prompts by the investigators were also used to allow the interviewee to discuss the topic further. Sociodemographic information was collected by the researchers using a questionnaire, and obstetric and neonatal information was extracted from medical records.

**Table 1:** Interview questions

Questions about skin-to-skin contact:
• Do you know anything about skin-to-skin contact? If so, can you please elaborate? • How do you feel when your baby comes into contact with your skin? • Have your feelings changed after touching your baby’s skin?
Questions about non-nutritive comfort sucking:
• Do you know anything about non-nutritive comfort sucking? If so, can you please elaborate? • How do you feel when your baby comes into non-nutritive comfort sucking? • Have your feelings changed after non-nutritive comfort sucking?
What are your opinions and suggestions for the NICU to carry out this project?

Before the interviews, participants were given the opportunity to ask questions. Before starting the study, the purpose and methods of the study were explained to mothers, as well as their right to end the interview at any time and for any reason without affecting their experience in the NICU, in addition to ensuring confidentiality of the data. All interviews were carried out by the first author (Liling Li), a charge nurse with experience in interviewing mothers during the perinatal period. Mothers were informed that the interviewer was not associated with infant care to encourage open and honest responses. The interviews were conducted in daytime and in a separate and quiet room for parents in the NICU, which lasted approximately 30–60 min, in Mandarin Chinese, and were transcribed verbatim, with all identifying information removed.

### Data analysis [20]

For the interview results, the inductive topic analysis method was used to identify, describe, and check the topics and data contained in the text, so as to conduct qualitative analysis. First, determine the concept or theme and name it to obtain the most basic meaning unit (code) in data analysis. The recorded data were converted into text and read repeatedly to get familiar with the data and determine the initial code. Secondly, the initial code was classified as potential topics, and the related code was placed under these topics. Thirdly, each topic was reviewed based on the generated code and the entire dataset. Finally, the topic was named and defined. NVivo 9 was used to organize codes and topics. The participant code was directly referenced to ensure anonymity. Data collection ended when no new information emerged from the interviews and data saturation was achieved. Two researchers conducted the data analysis. When there were disagreements, a third researcher to reach a consensus.

## Results

A total of 18 mothers, labelled A-R, were interviewed in this study. The mean admission age of preterm infants was 2.94 ± 1.21 h; the mean birth weight was 1239.44 ± 179.66 g, the mean gestational age was 28.74 ± 0.60 weeks. The mean weight and age on the day of the first skin-to-skin contact were 1666.11 ± 289.08 g and 31.39 ± 14.38d, respectively. The data of infants and their mothers are shown in Tables 2 and 3. These characteristics show that these babies were very vulnerable and many Chinese mothers did not feel they were able do skin-to-skin contact combined with non-nutritive comfort sucking with babies perceived to be fragile.

**Table 2:** Infant Characteristics

Number	Age of admission (h)	Birth weight (g)	Gestational age at birth (w)	Age of the infant on the day of interview (d)	Weight on the day of interview (g)
A	3	1280	27^+ 6^	42d	2090
B	3	970	27^+ 6^	47d	1570
C	2	1470	29^+ 1^	21d	1560
D	3	1240	28	48d	2000
E	3	1440	29^+ 1^	33d	1810
F	1	1390	30	5d	1280
G	2	1190	28^+ 5^	43d	2070
H	5	1340	30	23d	1560
I	1	1450	30	14d	1700
J	4	1390	28^+ 5^	38d	2040
K	4	1300	28	35d	1910
L	4	1395	29^+ 2^	14d	1390
M	5	910	28	48d	1530
N	3	950	26	47d	1500
O	2	1040	28^+ 2^	30d	1320
P	4	1160	28^+ 2^	44d	1950
Q	2	1265	29^+ 3^	23d	1510
R	2	1130	29^+ 3^	10d	1200

**Table 3:** Characteristics of the mothers

Items	value
Mother’s age (y)	32.2 ± 4.8
Mode of delivery, n(%)	
Cesarean section	7 (38.9)
Natural labor	11 (61.1)
Birth Count, n(%)	
One child	14 (77.8)
The second child	4 (22.2)
Education level, n(%)	
Undergraduate	8 (44.4)
Junior college	5 (27.8)
Junior middle school	1 (5.6)
Master	1 (5.6)
Secondary specialized school	3 (16.7)
Working status, n(%)	
Maternity leave	14 (77.8)
Work	4 (22.2)
Marital status, n(%)	
Married	18 (100)
Medical insurance, n(%)	
Yes	11 (61.1)
No	7 (38.9)
Round trip time(h)	2.2 ± 1.1

### Theme 1: alleviation of maternal anxiety and fear during mother infant separation

Early skin-to-skin contact combined with non-nutritive comfort sucking provides an opportunity to contact and bonding between the infant and mother during hospitalization of preterm infants when they were clinically stable. In the following descriptions by mothers, “cuddling” refers to simultaneous skin-to-skin contact and non-nutritive comfort sucking. The word was used to replace other terms the mothers may have used in describing their experience. Case B stated: “*I have not seen my baby for more than a month, and I miss him very much. The first time I saw the baby crying, I said to him when we were only the two of us, I finally saw you.*” Case H declared: “*it is hard to leave the maternity hospital and go home. I want to see him and know what his condition is. I want to find some psychological comfort. After cuddling, I feel some relief, and my heart doesn’t feel so bitter.*” Case I said: “*I am happier here than at home. I’m very anxious to see the hospital bill at home. When I see her here, I am not as anxious as I am at home. Yesterday, the doctor said she vomited. I don’t know how serious it was, so I am worried. Today, holding her, I saw a little vomit and felt it is OK.*“ Case J stated: “*Before I saw her, I always thought about her. My family never mentioned the baby in front of me, fearing that I would be sad. I feel relieved after I held her today.* “Case Q declared: “*at the beginning, every time I called to ask about the baby’s condition, his father and I were very afraid of hearing bad news. Now, we can see him every day and are no longer afraid when we call.*“ A mother said, “*before cuddling, my husband and I consulted the doctor every afternoon about the baby’s condition, and the grandparents even called to ask again. They were worried that our questions were not comprehensive enough. Although those questions were too boring to you, they tried to find some comfort through consultation. After cuddling, they never called again, I will share the baby with them through my eyes every day.*“

### Theme 2: Reshaping the maternal role

Admission of preterm infants to the NICU results in the separation of mother and infant, and the mother cannot participate in the care of preterm infants due to hospitalization, which prevents mothers from identifying their role. Case C stated: “*before cuddling, my family talked about my daughter, but I did not feel like a mother at all. After cuddling, we became closer. She depended on me, and I also depended on her. When she lies on me, I feel at ease. I am her world.*“ Case D claimed: “*before cuddling, I was confined. Motherhood was just a concept. Although I pumped milk every day, I did not feel like a mother. After the first cuddle, I appreciated motherhood.*“ Case K said: “*when I held her for the first time, she grabbed me. It was very real. I really felt like a mother.*“ Case P declared: “*when I breastfeed, I have a physical connection to her, and I naturally want to take care of her. I think it is the feeling of being a mother.*“ Case Q said: “*when the baby sucks on my breast, I feel more real as a mother. The pump is just a tool. It is a physical mother, and I become a spiritual mother when my baby sucks on my breast.*“

### Theme 3: Promotion of active breast pumping

The first food choice for very low birth weight infants is their own mother’s breast milk [21]. It is ideal for mothers to pump their breasts every 2–3 h to obtain breast milk when preterm infants are cared for in the NICU. With the baby not at their side, along with the difficulties of breast pumping, mothers often reject to pump breastmilk. Skin-to-skin contact combined with non-nutritive comfort sucking should be carried out as soon as possible to improve the mother’s willingness to breast pumping through psychological and physiological responses. Case B stated: “*before cuddling, I pumped breast milk without interest. My husband reminded me to pump breast milk every day. I was very upset. After holding the baby, my heart became soft. I took the initiative to find a way to increase the amount of breast milk, which was reported to the nurse. After cuddling, I feel that pumping breast milk is not a hard task. I hope she can feed more every day. I will provide more breast milk for her and do more things for her.* “Case D said: “*before cuddling, I pumped breast milk every 2 hours, which made me very tired and empty. I don’t know why. After holding the baby for the first time, my husband did not need to remind me to pump breast milk. I set the alarm clock by myself and got up to pump in the middle of the night. I want to keep my girl well and well grown.*“ Case H stated: “*there was very little breast milk pumped out before cuddling. After the first cuddling, I feel that the pumping is smooth. Every time I hold my baby, I feel my milk volume increasing. Now, the mood I feel when pumping is completely transformed.*“ Case P claimed: “*before cuddling, I struggled to pump breast milk every day. When I had to choose between two things, pumping breast milk wouldn’t be a priority. I felt that pumping breast milk was a task which had to be completed. After cuddling, I prioritize the pumping. I did not get up in the middle of the night before. Now I get up at 2–3 a.m. to pump more breast milk.*“

### Theme 4: Enhances the mother’s willingness to actively breast feed

In the NICU, tube or bottle feeding are the main feeding methods for hospitalized preterm infants. Because of the physiological characteristics of preterm infants and the lack of direct breastfeeding skills of mothers, breastfeeding failure often occurs after discharge. The most natural way of feeding is provided by the full contact between the preterm infant and the mother’s skin. With the guidance of the NICU’s lactation counselor, the preterm infant is gradually transitioned to breastfeeding after discharge. Case A stated: “*one day, the baby held my nipple and made a “tut tut” sound. She was very happy. It is cold to bottle feed and rewarding to breastfeed. I feel like she cannot live without me.*“ Case C said: “*I did not plan to breastfeed my baby. It’s troublesome to carry milk at work and have to wean later. After cuddling, I tried to breastfeed my baby. I found it was very good. It was different from the breast pump. His sucking made me feel very comfortable. I hope he can suck more.*“ Case D stated: “*after breastfeeding several times, I feel closer to my baby. I have a sense of responsibility and satisfaction. I feel that I am irreplaceable for her.*“ Case K claimed: “*when she sucks one of my breasts, the other is overflowing. My breasts are lactating! I feel so satisfied. When I get home, I still wonder if she will suck my breasts tomorrow.*“ Case L said: “*when the baby sucked my breasts for the first time, his small body curled up on me, and I felt full of happiness. He is my milkman. It was not smooth to pump breastmilk before cuddling. After breastfeeding, the amount of breastmilk increases suddenly, which is very helpful to me.*“ Case P said: “*after trying, I imagine that I will breastfeed my baby when he goes home. Before skin-to-skin contact and comfort sucking, I did not think I could breastfeed my baby by myself.*“

### Theme 5: Building the maternal confidence in baby care

In the early stage of hospitalization, the medical staff provide the opportunity for the baby to come into contact with the mother’s skin and comfort feed. During this process, the mother participates in the observation, feeding, and care, and experiences the baby’s growth process, which can improve the mother’s confidence in care after discharge. Case A stated: “*before cuddling, I thought I’d better wait for my baby to be 8 kg before leaving the hospital, because we don’t know how to take care of the baby at home. After cuddling, I spoke with the medical staff every day and I gradually learned to solve various problems my baby has. Now if I could, I’d rather take my baby and leave hospital as soon as possible because we can take better care of her.*“ Case C claimed: “*before cuddling, I worried that after leaving the hospital, I could not provide the same care my baby has had in the hospital. After all, she is a preterm baby. Now that I’ve held her for such a long time, I know how to hold her. I also know when she wants to eat. I feel I can take good care of her when she goes home.*“ Case L said: “*after cuddling, I am comfortable putting him directly on his father without help. My courage has grown, and I have learned how to deal with blood oxygen fluctuations and choking on milk. I’m not so flustered about when he comes home from the hospital. Now I don’t feel that stressed. Everything is fine.*“ Case Q said: “*before cuddling, I felt that I could not support him at home. After cuddling, I learned to feed and cuddle him. I think it’s very easy to start. After cuddling him, I consider him a member of my family. I don’t want him to stay here alone. I want him to go home early.*“

## Discussion

This study was carried out during the COVID-19 pandemic. Before entering the NICU, the mother was required to submit a negative nucleic acid test result no older than 72 h, have her temperature taken, and enter the NICU after answering a screening questionnaire. Only the mothers were invited into the NICU for skin-to-skin contact guided by the nurses.

Very low birth weight infants are transported to the NICU for medical evaluation and treatment immediately after birth. This immediate separation of mother and infant is a highly stressful event for parents and families of the infant [22]. In China, parents often use telephone consultations or appointments to obtain updates on their admitted preterm infants. Based on the concept of family integrated care, we are also developing the program involving the parents to take care their infants with healthcare staff. Because we have applied for the project about applying early skin-to-skin contact combined with non-nutritive comfort sucking in very low birth weight infants, all the medical staff in the NICU support the implementation of this project. Skin-to-skin contact combined with non-nutritive comfort sucking are ties to maintain the relationship between mother and infant, which makes mothers of preterm infants regain confidence in their role [23]. Because the mothers of preterm infants do not participate in infant care for a long time before discharge, they may perceive themselves as incompetent in taking care of preterm infants after discharge. Most of the published articles on skin-to-skin contact mainly focus on quantitative research, such as examining the growth and development of newborns, postpartum recovery of mothers, and breastfeeding promotion, all of which use data to reflect the intervention effects in China [24, 25]. However, the mental health of Chinese mothers of preterm infants, why mothers are not willing to actively pump breastmilk when mother and baby are separated, and why they are more willing to leave the baby in the hospital rather than bringing them home are all worthy of attention. In this study, psychological interviews were used to explore mothers’ experiences caused by the lack of mother-baby contact, and the information that cannot be obtained by quantitative research such as the change of the mother’s mental state and behavior as well as the transformation of her perceived role after skin-to-skin contact combined with non-nutritive comfort sucking. The psychology and behavior of mothers of preterm infants run through the whole process of care for preterm infants. In this study, preterm infant admission to the NICU after birth not only had an impact on parents’ emotional responses, but also caused anxiety to the whole family. During the interview, we learned that nurses’ behaviors and words also changed the mothers’ psychology before and after holding the infants. Psychological interviews provided the mothers an opportunity to talk about the situation, through communication to understand the mothers’ psychological changes before and after skin-to-skin contact, to identify the reasons behind the parents’ behaviors and to provide clues for the improvement of medical services.

The NICU’s medical staff are disseminators of knowledge and promoters of practice in the care of preterm infants. Among the 18 interviewees, only one mother was informed about skin-to-skin contact through the media before skin-to-skin contact. When a mother is informed that she can enter the NICU to hold her baby, most of the surprise comes from the fact that she can see her baby. All mothers were nervous while cuddling their infants for the first time. It was shown that 70% of skin-to skin-contact and comfort sucking in the NICU are initiated by nurses [26]. The parents’ knowledge mainly comes from nurses in the NICU, friends and relatives with preterm birth experience, and internet resources. The mothers in this study gained knowledge for care during cuddling and witnessed changes in their infants as well as the regulation of their own emotions in practical interaction. Early skin-to-skin contact and non-nutritive comfort sucking have been shown to support the development of preterm infants, but their implementation in the NICU needs the support and approval of the medical staff [27, 28]. In the study, the mean weight in preterm infants at first skin-to-skin contact with their mothers was (1666.11 ± 289.08) g, and the minimum weight was 1200 g. In the absence of encouragement, mothers expressed fear and worry. The concept of skin-to-skin care is to provide preterm infants with early, sustained, prolonged skin to skin contact with mothers. In this study, all mothers completed early and continuous skin-to-skin care with the guidance and encouragement of nurses. The NICU staff, especially nurses, are caregivers for hospitalized preterm infants, and the disseminators of knowledge in caring for newborns. Encouragement and support from NICU nurses can reduce fear in mothers and allow early skin-to-skin contact combined with non-nutritive comfort sucking [29, 30].

### Strengths and limitations

The strength of this research is that it deeply explores the psychological changes of mothers when they can enter the NICU and carry out special care mode of skin-to-skin contact and non-nutritive comfort sucking, which is different from the previous normalization mode of maternal and infant separation in China’s NICU. The psychological feelings cannot be obtained by quantitative study. It also provides the evidence for the benefits of family integrated care practice in NICUs.

This was a single-center study carried out in China during the COVID-19 pandemic, and due to the culture of confinement in China, not all eligible mothers of premature infants were enrolled in the study during the period. Participants included were more active and more easily motivated to talk about their experiences and feelings, it should be expanded to carry out in multi-center and include more participants with different culture background in the future.

## Conclusion

Skin-to-skin contact combined with non-nutritive comfort sucking in the NICU can not only alleviate maternal anxiety and fear due to mother - infant separation, enhance the identity and responsibility of the mother’s role, reshape the role of mothers, but also promote breastfeeding and improves maternal confidence in baby care. And it will be beneficial to promote the establishment of oral feeding in preterm infants.

## Data Availability

All data are available in the manuscript. The corresponding author can be contacted for additional information if required.

## Abbreviations

KMC: Kangaroo mother care
NICU: The neonatal intensive care unit.
